# Microstate ERP Analyses to Pinpoint the Articulatory Onset in Speech Production

**DOI:** 10.1007/s10548-020-00803-3

**Published:** 2020-11-08

**Authors:** Anne-Lise Jouen, Monica Lancheros, Marina Laganaro

**Affiliations:** Faculty of Psychology and Educational Science (FPSE), University of Geneva, 28 Boulevard du Pont d’Arve, 1205 Geneva, Switzerland

**Keywords:** Speech production, EEG, Microstate ERP, Response-locked ERPs, Articulatory onset to acoustic onset interval (AAI)

## Abstract

The use of electroencephalography (EEG) to study overt speech production has increased substantially in the past 15 years and the alignment of evoked potential (ERPs) on the response onset has become an extremely useful method to target “latest” stages of speech production. Yet, response-locked ERPs raise a methodological issue: on which event should the point of alignment be placed? Response-locked ERPs are usually aligned to the vocal (acoustic) onset, although it is well known that articulatory movements may start up to a hundred milliseconds prior to the acoustic onset and that this “articulatory onset to acoustic onset interval” (AAI) depends on the phoneme properties. Given the previously reported difficulties to measure the AAI, the purpose of this study was to determine if the AAI could be reliably detected with EEG-microstates. High-density EEG was recorded during delayed speech production of monosyllabic pseudowords with four different onset consonants. Whereas the acoustic response onsets varied depending on the onset consonant, the response-locked spatiotemporal EEG analysis revealed a clear asynchrony of the same sequence of microstates across onset consonants. A specific microstate, the latest observed in the ERPs locked to the vocal onset, presented longer duration for phonemes with longer acoustic response onsets. Converging evidences seemed to confirm that this microstate may be related to the articulatory onset of motor execution: its scalp topography corresponded to those previously associated with muscle activity and source localization highlighted the involvement of motor areas. Finally, the analyses on the duration of such microstate in single trials further fit with the AAI intervals for specific phonemes reported in previous studies. These results thus suggest that a particular ERP-microstate is a reliable index of articulation onset and of the AAI.

**Electronic supplementary material:**

The online version of this article (10.1007/s10548-020-00803-3) contains supplementary material, which is available to authorized users.

## Introduction

Producing an utterance is a complex process, involving multiple systems and mental operations in order to transform an abstract code into articulated speech. This transformation requires cognitive as well as motor processing (Levelt et al. 1999). The “latest” stages of speech production -when a linguistic message is transformed into a motor code- have received less attention than linguistic encoding processes, in particular in the neuroimaging literature. The cortical regions involved in the different stages of speech production are actually relatively well-established (Papoutsi et al. 2009; Mugler et al. 2018), but the study of their spatiotemporal dynamics remains poorly investigated, probably because of the methodological challenges it involves. Characterizing and isolating motor speech planning and execution requires an alignment of event-related potentials (ERPs) to the speech onset (Laganaro and Perret 2011; Riès et al. 2013), which raises the issues of motor artifacts (Ganushchak and Schiller 2008; Ganushchak et al. 2011; Vos et al. 2010; Riès et al. 2011; Porcaro et al. 2015; Ouyang et al. 2016) and of the point of alignment of ERPs (Fargier et al. 2018). The most common strategies consist in aligning ERPs to the stimulus eliciting the produced utterance (written stimulus or picture) and analyzing a time-period not extending beyond the shortest response latency to include only artifact-free periods. However, to investigate “later” (speech) encoding processes during utterance production, ERPs should be analyzed closer to the response, as pointed out by Riès et al. (2013), “*the core aspect of language production is not perception but action. (…) the most relevant question may therefore not be how long after the stimulus brain events happen, but rather how long before the production act do they occur*”. In the past 10 years, response-locked approaches have been developed (Laganaro and Perret 2011; Riès et al. 2013; Van der Linden et al. 2014; Laganaro 2014) and have become an extremely useful approach to target later stages of speech production.

Yet, the response-locked ERPs raise another issue, namely where (on which event) to place the point of alignment. Indeed, the usual ERP alignment point used in speech production studies, the acoustic vocal onset of speech (onset of energy in the acoustic signal), does not correspond with the articulatory onset of motor execution. This gap between articulatory and vocal onsets is called by some authors the “articulatory onset to acoustic onset interval” (AAI,^1^ Kawamoto et al. 2008) and is known to depend on the properties of the phonemes.

### Impact of the Initial Phoneme on the Onset of Acoustic Energy/Articulation

The fact that articulation may start several tens of milliseconds before vocal onset has been described a long time ago by phoneticians (Halle et al. 1957; Bell-Berti and Harris 1981; Brooker and Donald 1980). In particular a 50 to 100 ms longer AAI has been shown for voiceless stop consonants relative to non-plosive ones (see Kawamoto et al. 2008). Yet, the onset of acoustic energy in verbal responses has continued to be used as an index of response latency (acoustic latency) to investigate the processes that occur prior to response execution (Kawamoto et al. 2008) and as the point of alignment of response-locked ERPs.

The asynchrony between articulatory and acoustic onsets and the temporal aspects of motor execution during speech production have been formalized experimentally about ten years ago using delayed-production tasks and varying the onset phoneme. Delayed production has been used to study separately motor speech preparation and execution in acoustic studies (Rastle et al. 2005; Kawamoto et al. 2008) and in neuroimaging studies (Chang et al. 2009; Mock et al. 2011; Tilsen et al. 2016; Lancheros et al. 2020). The idea behind this task is that the delay allows participants to retrieve and prepare their response and that the differences in the acoustic onset observed across items can only be due to the articulatory to acoustic properties of the onset phonemes. The acoustic studies (Rastle et al. 2005; Kawamoto et al. 2008) revealed that the properties of the first phoneme have a strong effect on production latency: indeed, the authors observed a varying time lag between the onset of motor execution and the onset of acoustic energy across initial consonants. The study by Mooshammer et al. (2012), combining both acoustic and articulatory measures (electromagnetic articulography EMA), directly showed that this varying time lag was due to the misalignment of articulatory and vocal onsets. Indeed, the authors also reported longer acoustic reaction times (acoustic RTs) for words with onset stop consonants (/p,t,k/) relative to fricatives (/s/), whereas they observed only minor effects for the results based on articulatory measurements (articulatory RTs). These results clearly indicated that the articulatory initiation times were very similar for fricative and stop consonants whereas they were dissimilar for the vocal onset, resulting in differential AAI.

Taken together, these results show that the gap between the onset of acoustic energy and the onset of the articulatory movement highly depends on the phonemes features, which makes the onset of acoustic energy clearly not ideal to determine the beginning of articulation.

Furthermore, the articulatory onset of motor execution has received different definitions, making even more difficult the delimitation of AAI: if the articulatory onset generally corresponds to a detectable change of position of the articulators or to detectable muscle activity (not accompanied by detectable movement), the onset of motor execution has also been conceptualized as *“the moment at which the cognitive plan for speech is delivered to the speech execution system, initiating coordinated movement of the articulators*” (Rastle et al. 2005) rather than an actual muscle activity. According to Rastle’s definition, the onset of motor execution would not correspond to an observable physical event and thus, it would not be possible to obtain a direct measurement of the execution–acoustic interval (EAI, aka AAI).

This lack of a clear definition of the onset of motor execution also explains why delimitating the AAI can be hard with traditional methods -such as electromyography (EMG) or video- and how EEG could be used instead, as reviewed in the following section.

### Limitations of Articulation Onset Detection Techniques for the EEG Study of Speech Production

There are nowadays different tools to obtain articulatory measures (electromagnetic articulography EMA, electropalatography EPG, MRI…) which are also able to give a good insight into the start of articulatory execution. However, most of these investigation techniques are quite invasive, not adapted for experiments with many participants and/or not compatible with the acquisition of EEG signals.

Although video recording may represent a simple and non-intrusive way to track articulatory movements, it would only allow to detect lips/jaw movements whereas palatal/velar ones would be much harder to identify. Video recording would also miss the voicing phenomena (i.e., vocal-fold vibration) occurring without any preceding lip-muscle movement (Van der Linden et al. 2014).

The most widely used technique is probably electromyography (EMG), but it also presents several methodological caveats (Van der Linden et al. 2014). A first issue is related to which muscle activity should be measured, given that speaking involves moving more than 100 muscles in the lips, tongue and vocal folds. EMG activity is not informative either depending on the different effectors (e.g.: vocal-fold vibration and velar movements are not detected), hence the use of face EMG may provide highly variable results according to the phonemes produced. EMG signals have also been shown to vary across speakers, speaking styles and even across recording sessions of the very same speaker (Jou et al. 2007; Wand et al. 2009). Finally, unlike manual EMG, speech-related EMG often results in multiple bursts within the period of interest (notably incidental muscular activity such as prephonatory breath) making any attempt to place the point of fractionation/alignment very difficult (Van der Linden et al. 2014). All these drawbacks prevent the use of EMG signals to lock ERPs in the EEG investigation of speech, which is why other solutions should be sought.

The work by Fargier and collaborators (2018) represents a first piece of evidence that EEG signal may be used as a marker of articulation onset, being more accurate than the vocal onset. The authors showed how the nature of the first phoneme influences the point of alignment of ERPs and consequently the ERP signal, demonstrating a consistent shift of about 40 ms on the ERPs locked to the vocal onset between a voiced and an unvoiced bilabial stop consonant (/b/vs/p/), both in gamma band oscillations and in the global electric field at scalp.

Hence, it is well known that different phonemes have different articulatory-to-acoustic onset delays and that such varying AAI impacts the EEG/ERP signal aligned to the vocal (acoustic) onset. For the reasons exposed above, there are no straightforward approaches to take the phoneme-specific AAI into account when analyzing response-locked ERPs.

Here we aim at determining whether and how topographic analyses can pinpoint the delay between onset of articulation and vocal onset, taking advantage of high temporal-resolution electroencephalographic/evoked potential (EEG/ERP) in a delayed production task. In particular, we explored if a specific electrophysiological activity at scalp (microstate) could be associated with the AAI. We reasoned that if the duration of a specific microstate locked to the acoustic onset of different phonemes (voiceless stops and fricatives) varies according to the articulatory-to-acoustic onsets -which are known for these phonemes-, such microstate would likely correspond to the electrophysiological signature of the AAI.

## Material and Methods

### Participants

26 French speakers with normal or corrected-to-normal vision participated in the experiment (10 men; mean age: 24.8, SD = 4.7 years). All subjects gave their informed consent to participate in the study, approved by the local ethics committee, and were paid for their participation. Only participants completing the task with an accuracy > 75% were retained. Of the 25 subjects that reached this criterion, three were excluded from the analysis due to over-noisy EEG recordings, thus leaving 22 participants for the analysis.

### Material

The stimuli were 112 monosyllabic pseudowords starting with consonant clusters (CCV),^2^ beginning with stop consonants (/p/, /t/, /k/), and the corresponding CCCV resulting from the addition of a fricative (/s/) onset- all the other phonemes being the same- (example: /pre/, /trɛ̃/, /kRa/, matched with /spre /strɛ̃/ /skRa/).

The initial consonants were chosen because of their dissimilarity on the AAI: vocal and articulatory onsets are very close for fricatives whereas they are not for stimuli starting with stop consonants, which makes their precise start onset unclear (Ouyang et al. 2016). The stops and fricatives used in this study are similar for some articulatory features (voicelessness, oral and central consonants, airstream mechanism) but they differ on the manner and place of articulation (/p/ = bilabial, /t/ = dental/alveolar, /k/ = velar, /s/ = alveolar). The choice of the initial consonants was also compelled by the fact that we aimed to use French syllables existing in initial position, from which we could build CCCV -which was only possible with the /s/-beginning fricatives-.

### Procedure

Participants sat in front of a computer screen (approximately 70 cm) in a sound-proof dimly lit room. The experimental software E-prime (version 2.0, Schneider et al. 2002) was used for stimuli presentation and data collection. First, the participants were familiarized with all the pseudowords presented in a random order: they had to repeat them overtly after each presentation (presentation was both auditory and visual). Then, participants underwent a training phase on the delayed production task (five warm-up filler trials, repeated if necessary) accompanied by the experimenter who explained the task. Finally, the experimental phase started. The task was divided in three blocks to allow participants two brief breaks in between.

**Fig. 1:**
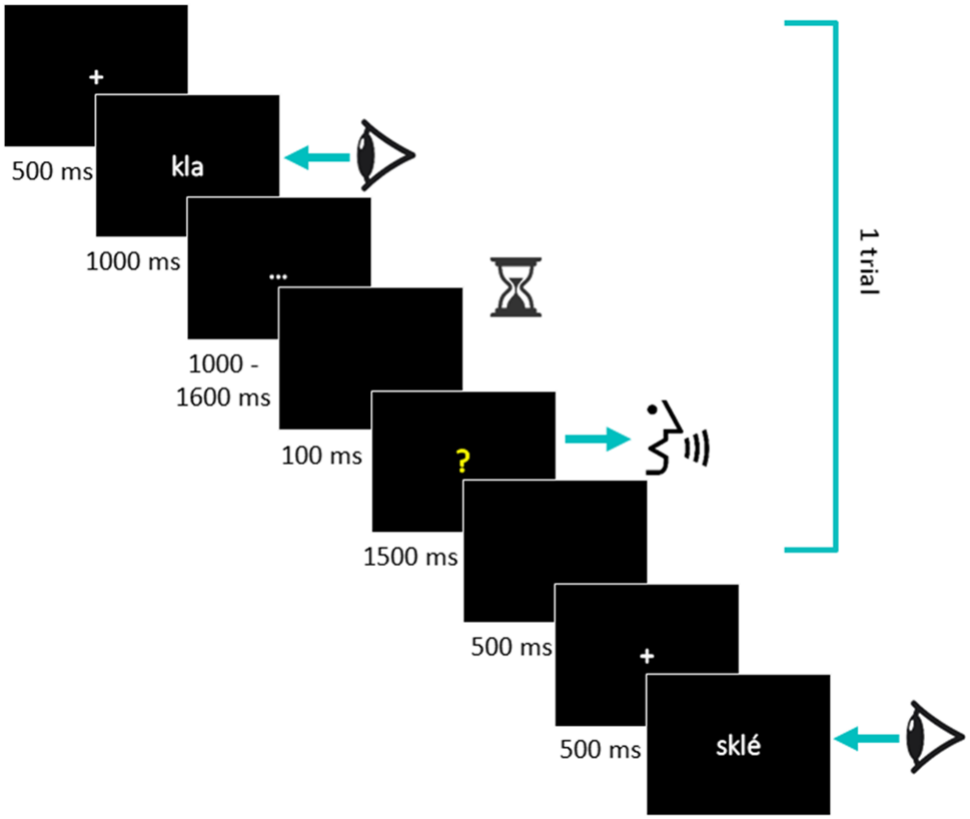
Experimental procedure. Participants responded when the cue “?” appeared on the screen. Acoustic latencies were calculated from the apparition of the “?” to the vocal response onset

A trial started with a fixation cross presented for 500 ms (in white on a black screen), then a written pseudoword appeared on the screen and remained for 1000 ms, followed by “…” in white, which randomly lasted between 1000 and 1600 ms in steps of 300 ms. A variable delay was used so that participants could not anticipate the response cue (see Laganaro and Alario 2006 for the rationale behind the duration of the chosen delays). Only filler items were presented at the shortest delay (1000 ms) and the corresponding answers were not included in the analysis. Participants were instructed to wait silently until the response cue appeared. After a brief blank screen (100 ms), the response cue (a yellow question mark) remained on the screen for 1500 ms indicating that participants had to repeat the target stimulus as fast and accurately as possible (See Fig. 1). For filler items “…” appeared in yellow instead of the question mark and participants only had to wait until the next trial.

All stimuli were presented twice throughout the task (except a unique presentation for the filler items, 252 stimuli in total), once in each of the two delays (1300 or 1600 ms). Items were pseudo-randomized (two different lists) such that the same stimulus was not presented consecutively and the same delay was not presented for more than three consecutive trials.

### EEG Acquisition

The EEG signal was recorded continuously using the Active-Two Biosemi EEG system (Biosemi V.O.F., Amsterdam, Netherlands) with 128 channels covering the entire scalp. Signals were sampled at 512 Hz (filters: DC to 104 Hz, 3 dB/octave slope). The custom online reference of the system is the common mode sense–driven right leg (CMS-DRL).

### Preprocessing and Analyses

#### Alignment to the Acoustic Onset

**Fig. 2:**
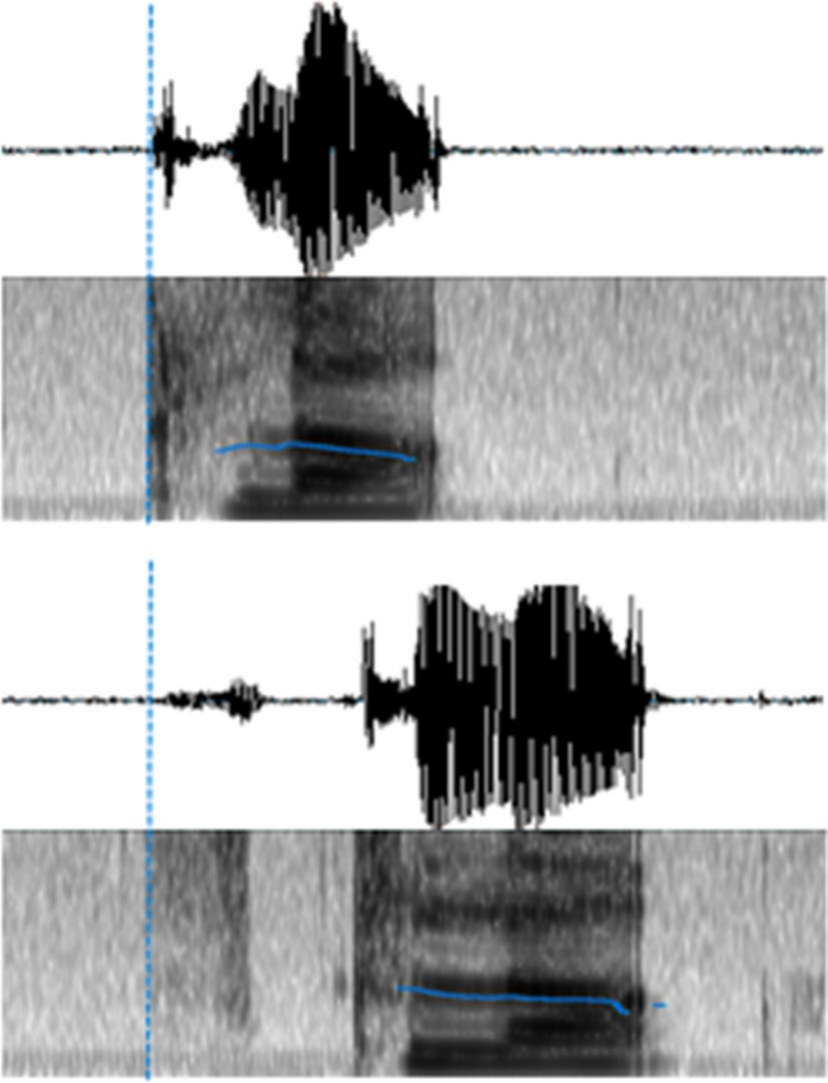
Time course of acoustic energy for k-initial items (/kla/ on the top) and s-initial items (/skla/, on the bottom), with respective vocal onsets marked

The digitized responses were manually checked with a speech analysis software (CheckVocal 2.2.6, Protopapas, 2007) to identify correct responses and acoustic onsets (from the question mark to the vocal onset, vocal RT hereafter). No-responses, wrong responses (i.e. production of a different stimulus than the target), hesitations and/or auto-corrections were considered as errors. For voiceless stop consonants, the airflow has first to be trapped behind the oral constriction by the supraglottal articulators, resulting in an intra-oral pressure. The release of this pressure triggers acoustic energy, so that the onset of acoustic energy corresponds to the time point when the supraglottal articulators stop maintaining the oral constriction and begin to move from their current configuration to the target position of the next segment. For fricatives, the air flow through the oral tract passage is never completely blocked and acoustic energy is generated with the air moving through a narrow oral constriction. Thus, the onset of acoustic energy for fricatives invariably corresponds to the moment when air begins to be channeled through this oral constriction. Because of these differences, the acoustic energy onset corresponds to the burst release for the voiceless stop consonants whereas for voiceless fricative consonants, it corresponds to fricative acoustic signature on the spectrogram. Typical examples of acoustic onsets are presented in Fig. 2.

#### EEG Data Pre-Processing

All the pre-processings were computed for each participant using the Cartool software (Brunet et al. 2011). Offline, EEG was high and low-pass filtered (0.1–30 Hz; 2ndorder a causal Butterworth filter with 212 dB/octave roll-off), notch-filtered (50 Hz) and ERP epochs were extracted. Epochs of 150 time-frames (i.e. ~ 293 ms) were extracted, time-locked to the onset of the vocal (acoustic) response (i.e., backward = response-locked^3^). Each ERP epoch was visually inspected; epochs contaminated by eye blinks or other noise artifacts were rejected and excluded from averaging. Only trials with correct responses, valid RTs and uncontaminated data were included in the analysis (in average 80% of the total number of epochs). Electrodes presenting artifacts were interpolated using 3-D splines interpolation (Perrin et al. 1987). The average of interpolated electrodes by participant was 11% (max = 16%; or up to 20 of the 128 electrodes). ERPs were averaged per participant and per condition (stops vs fricatives) and single epochs ERPs were also used in the analyses.

#### Microstate Analyses

The aim of microstate analyses is to determine whether conditions differ in global electric fields (e.g., Michel et al. 2009; Michel and Murray 2012). More precisely, a global topographic ERP pattern analysis, called a spatio-temporal segmentation, is performed on the group-averaged ERPs to determine topographic differences across conditions and statistically validate them in the ERPs of single participants. In the present study, these analyses were used to compare EEG topographies associated with our conditions of onset consonants. Changes in electric field take place when the underlying generator configuration has changed and differences in underlying generator suggest activation of different brain networks. Given that the topography of facial (lip, jaw, eyebrows…) or tongue movements have been described (Vanhatalo et al. 2003; Goncharova et al. 2003; McMenamin et al. 2011; Ma et al. 2012; Georgieva et al. 2018), we expect to find that the onset/duration of such microstate depends on the specific AAI for our onset consonants.

All the analyses were computed with the Cartool software (Brunet et al. 2011). The first step of the analyses consisted of a topographic analysis of variance, a non-parametric randomization test aimed to compare the global dissimilarity between two electric fields. This analysis, called “TANOVA” (Murray et al. 2008) was carried out to determine time point by time point to what extent the topography of the ERPs differs across conditions (stop versus fricative onsets), by focusing on the global dissimilarity index (GDI) which is a quantification of these topographic differences between two electric fields independently of their strength (Lehmann and Skrandies 1980). The data were permuted by re-assigning randomly the topographic maps of single subjects to the different conditions. The GDI of these random group-averaged ERPs was compared time point by time point with the values of topographic dissimilarity of the actual conditions in order to determine the likelihood of obtaining a higher GDI value than the one actually obtained. In the present study, this analysis was conducted with an alpha set to 0.01 and a time period criterion of 10 ms of consecutive significant difference.

Then the spatio-temporal segmentation was performed on the group-averaged ERPs from each condition. This procedure corresponds to a segmentation of ERPs in periods of stable global electrophysiological pattern at scalp (i.e. topographic maps or ERP microstates) by compressing the variability of ERPs in a series of template maps, summarizing the data and used to determine which topographic template best explains participants’ ERP responses to each experimental condition (Pascual-Marqui et al. 1995; Michel & Murray 2012). Statistical smoothing was applied to remove temporally isolated topographic maps with low explanatory power, a given ERP topography had to be present ≥ 10 ms in accordance with the criteria for the TANOVAs (Brunet et al. 2011; Murray et al. 2008).

Finally, topographic maps observed in the group-averaged data were statistically tested by comparing each map templates with the moment-by-moment scalp topography of individual ERPs (“fitting” procedure). A first fitting was computed to determine how well each topographic template map observed on the grand-averaged ERPs explains single participant responses for each condition. Each data sampling point was labelled according to the template map with which it best correlated spatially, giving as output variable the map duration (number of Time Frames, TF^4^) in each individual data. These data were used for the statistical comparison of the topographic differences between the different consonantal conditions. A second fitting was applied to the single trials/epochs (N = 3856) in order to further analyze whether differences across conditions were observed at the single trial level and, particularly, if differences when observed between places of articulation of the three stop consonants.

#### Source Localization

Brain electrical sources were determined for the identified microstates locked to the acoustic onset of stops and fricatives, from the individual average ERPs (two source localization analysis were produced separately: one based on the individual average ERPs of the stops consonants and the other based on the fricatives ones). The procedure described in Michel and Brunet (2019) was followed with the Cartool software (Brunet et al. 2011). The head model -the model for which the EEG forward solution is calculated- was constructed from the MNI average brain (MNI 152, Montreal Neurological Institute, Montreal, Canada) and co-registered with a template file containing the location of each electrode of the biosemi cap. The LSMAC (Locally Spherical Model with Anatomical Constraints) Lead Field was then calculated, providing the matrix from which the inverse problem was solved, using the linear distributed source model LORETA (Low Resolution Electromagnetic Tomography, Pascual-Marqui et al. 1994).

#### Statistical Analysis

RTs and EEG/ERP microstate data were fitted with mixed models (Baayen et al. 2008) with the R-software (R-project, R-development core team 2005). For behavioral data and for EEG/ERP microstate fitting data in single epochs (see below), the models were computed respectively with vocal RTs or map duration (TF) as dependent variables, the mode of articulation (fricatives versus stops) and place of articulation of the stop consonants (whether initial or following the fricative /(s)p/-, /(s)t/-, /(s)k/-) as fixed factors, as well as subjects and items as random factors.

## Results

### Behavioral Results

Production accuracy was high for both conditions (92% for the stops and 89% for fricatives). The production latencies for each onset condition are reported in Table 1.

**Table 1:** Mean response latencies (in milliseconds) and standard deviation for fricative and stop onset according to the mode of articulation of the stop consonants

Stop onset		Fricative onset	
/k/-	610.88, (151.94)	/sk/-	529.76, (149.32)
/p/-	628.80, (156.12)	/sp/-	525.47, (146.65)
/t/-	623.33, (155.85)	/st/-	538.12, (147.67)

Mean response latencies (RTs) for stop and fricative onsets were respectively 620.87 ms and 531.83 ms, resulting in a difference of ≈ 90 ms. The linear mixed model revealed a main effect of the mode of articulation, with fricative onsets being produced significantly faster than the voiceless stops (F(1, 222.31) = 426.32, p < 0.001) and a tendency for an interaction between mode and place (F(2, 222.24) = 2.59, p = 0.07).

The split of the data between mode of articulation confirmed an effect of place of articulation only for the initial stops (F(2, 109.47) = 3.15, p < 0.05 (for stops following fricative onsets: F(2, 112.1) = 1.85, p = 0.16).

The contrast results revealed significant differences on RTs across the three places of articulation of the stop onset consonants. RTs were faster for /k/ onsets as compared to /p/ (t(109.74) = 2.43, p < 0.05; β = 18.84, SE = 7.74) and they tended to be significantly faster as compared to /t/ (t(108.82) = 1.77, p = 0.07; β = 12.66, SE = 7.15). No significant differences were observed between phonemes /p/ and /t/ (t(109.90) < 1).

### ERP Results

#### TANOVA and Topographic Pattern Analysis

**Fig. 3:**
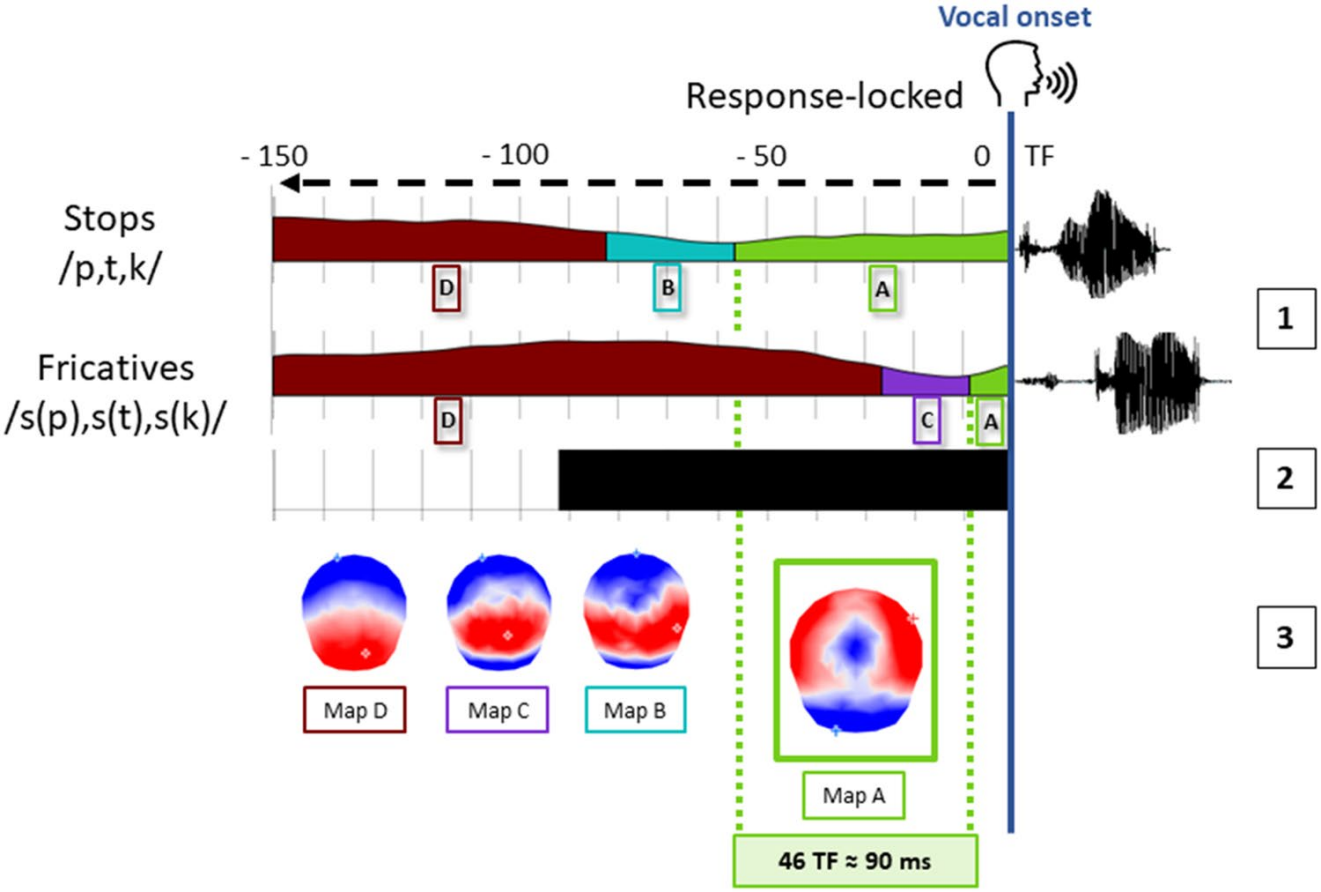
(1) Temporal distribution of the topographic maps revealed by the spatio-temporal segmentation analysis displayed with different colors under the mean GFP from the grand average of each condition locked to the vocal onset, with the corresponding template maps A, B, C and D (3). (2) Time-windows of significant TANOVA are displayed with the black bar

Pairwise TANOVAs on the response-locked ERPs revealed significant differences across conditions in the 90 TF (≈180 ms) before the vocal onset (see Fig. 3).

The spatio-temporal segmentation applied on the grand-averaged data for each condition revealed four different electrophysiological template maps for the response-locked ERPs (see Fig. 3), accounting for 95.6% of the variance.

The maps A to D were fitted from -150 to 0 TF before the vocal onset. The results from the fitting in the individual ERP signals revealed significant differences in duration TF for maps A and D. These results are reported in Table 2. Map A yielded higher longer duration for stop consonants relative to fricatives. The opposite result was observed on map D. Given that the analysis was computed on fixed time-windows, the results on Map D are the consequence of the shift of the previous maps starting from the alignment point. Maps B and C did not differ across conditions.

**Table 2:** Mean duration (in number of TF and ms) in the individual ERPs for each of the four topographic maps observed on the grand-averaged ERPs (response-locked analysis)

		Duration (Number of TF and equivalent in ms)				
		Mean	Std. error	df	t	p value
Map A	Stop fricative	***56.19 ***(≈ 110 ms) ***10.52 ***(≈ 20 ms)	***7.99*** ***2.55***	***21***	***2.09***	***.00019***
Map B	Stop fricative	8.19 (≈ 16 ms) 6.48 (≈ 13 ms)	2.86 2.61	21	2,09	.63
Map C	Stop fricative	11.57 (≈ 23 ms) 16.67 (≈ 33 ms)	4.33 4.65	21	2.09	.13
Map D	Stop fricative	74.05 (≈ 145 ms) 110.95 (≈ 217 ms)	8.32 7.42	21	2.09	.0011

#### Fitting in the Single Trials

The mixed model was run on the duration of the map of interest, Map A, highlighted in the previous analysis (yielding longer duration for stop consonants relative to fricatives, similar to the differences observed on vocal latencies), using the factors described in the statistical analysis paragraph.

The results of the fitting in single epochs are reported in Table 3.

**Table 3:** Mean duration (in TF and equivalence in ms) and standard deviation of Map A fitted in the single trials for fricative and stop onset according to the mode of articulation of the stop consonants

Stop onset		Fricative onset	
/k/-	51.3 ≈ 100 ms, (17.3)	/sk/-	43.0 ≈ 84 ms, (14.7)
/p/-	67.7 ≈ 132 ms, (16.7)	/sp/-	39.7 ≈ 77 ms, (18.9)
/t/-	62.0 ≈ 121 ms, (21.2)	/st/-	42.4 ≈ 83 ms, (14.3)

The results of the single trial analysis indicated a significant effect of the mode of articulation (F(1, 105) = 247.82 p < 0.001) confirming the shorter duration for /s/ onset (41.8 TF ≈ 81 ms) relative to stop onsets (61.9 TF ≈ 121 ms), and an interaction between mode and place of articulation (F(2, 105) = 9.29, p < 0.001). The split of the data between mode of articulation confirmed an effect of place of articulation only for initial stops (F(2, 53) = 9.68, p < 0.001 (for stops following fricative onsets: F(2, 52) = 1.27, p = 0.3).

The contrast results revealed significant differences in terms of duration across the three places of articulation of the stop onset consonants. The duration of map A was significantly shorter for the /k/ onsets as compared to /p/ (t(114.67) = 5.23, p < 0.001; β = 10.77, SE = 2.06) and to /t/ (t(111.86) = 2.98, p < 0.01; β = 5.65, SE = 1.89) as well as for phoneme /p/ compared to /t/ (t(113.98) = 2.60, p < 0.05; β = 5.11, SE = 1.96).

Hence, large differences (46 TF ≈ 90 ms on the grand averages and 20 TF ≈ 40 ms on single trials) were observed between voiceless stops and fricatives on the last topographic map preceding the point of alignment to the vocal onset, but longer duration of map A also characterized the signal preceding the production of /p/ relative to /k/ and /t/.

#### Source Localization Results

**Fig. 4:**
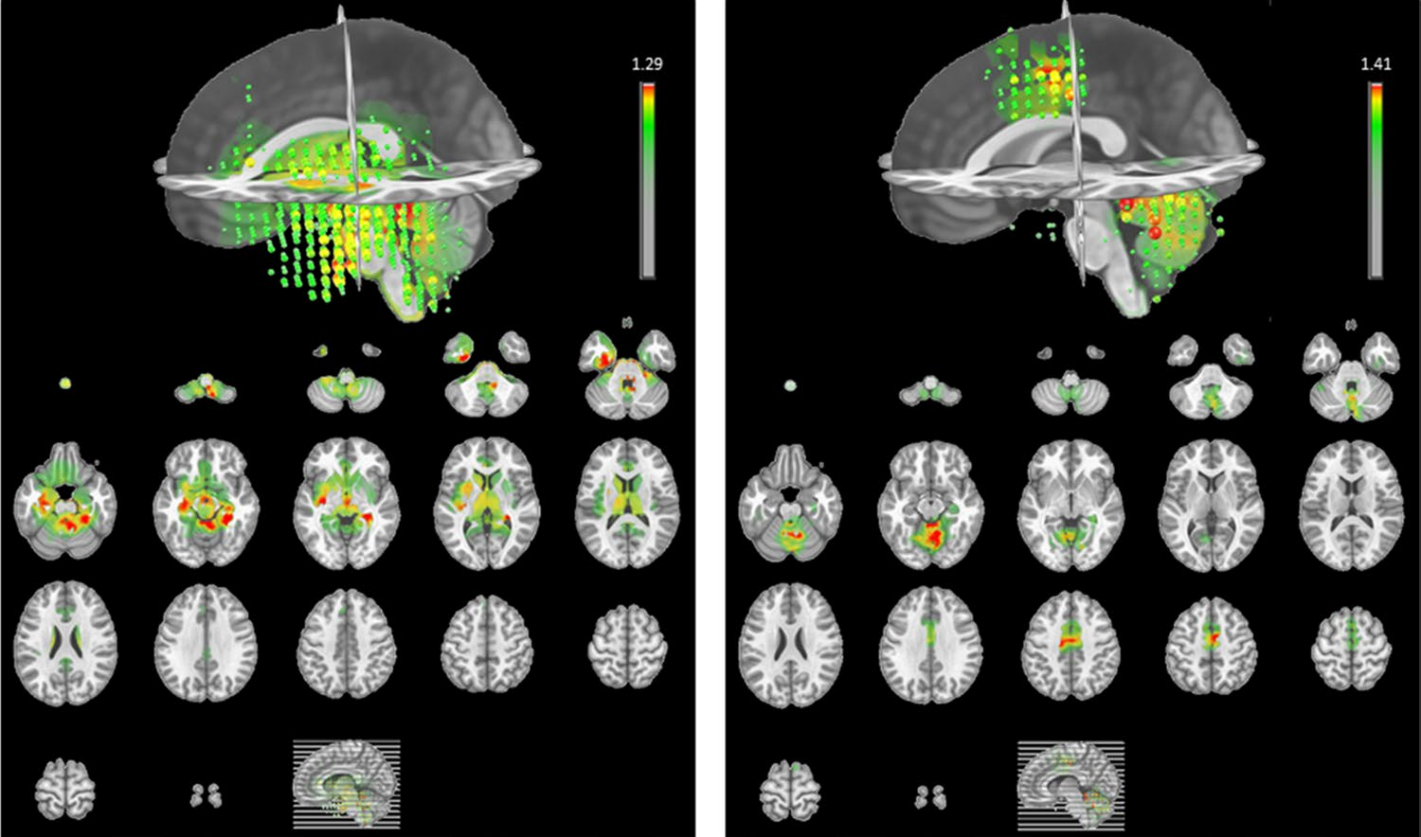
Illustration of the results of source localization (top in 3D and bottom as transverse slices), corresponding to the time-windows of maps D (left, −150 to −100 TF) and A (right, −50 to 0 TF)

We compared the results obtained from the source localization analysis for the two time-windows corresponding to the maps D and A (respectively −150 to −100 TF and −50 to 0 TF locked to the vocal onset, see Fig. 4). They revealed an activity mainly located in the left temporal and bilateral cerebellar regions for map D. A similar bilateral cerebellar activation was observed for map A as well as a specific activation for this map, in the premotor cortex in both hemispheres.

## Discussion

In this study, we aimed to evaluate the use of EEG/ERP microstate analysis to pinpoint the articulatory onset to acoustic onset interval (AAI) in speech production studies.

The first result highlighted by this work was a clear asynchrony between the onset of acoustic energy arising from a verbal response (vocal RT) and the time-distribution of the ERP microstates aligned to the vocal onsets. Behavioral analysis on production latencies identified by the vocal onset revealed that the /s/-pseudowords were initiated approximatively 90 ms faster than the /p/, /t/, /k/-onsets, which is consistent with the literature on the acoustic properties of our target consonants. As in a delayed production task there are a priori no reasons for RTs to be different across speech items (the delay allowing participants to retrieve and prepare their response), these differences are only due to the AAI of the different onset phonemes. The microstate ERP analyses provided an interpretation of such differences showing a shift of microstates across conditions likely reflecting the misalignment of articulatory and vocal onsets. Huge differences have also been observed in the TANOVAs in a period of time running from 180 ms to the vocal speech onset, in line with the mismatch in the distribution of the maps. Indeed, the spatio-temporal segmentation revealed global topographic ERP patterns (maps) similar in all onset conditions with the specific microstate “A” shifted on average of about 90 ms for the /s/-onsets relative to /p/, /t/, /k/ on the individual ERPs (a smaller difference in the same direction was also observed in the single trials analysis) This topographic map is very likely associated with articulatory movements as further discussed below.

### EEG Topographic Signature of Articulatory to Vocal Onset

Before discussing the main results about the topographic map preceding the vocal onset, we will briefly discuss the other results. As already stated above, the statistical differences on the topographic map labelled “D” in Fig. 3 are related to the fact that this map is probably not fully represented in the fixed time window of 150 time frames (~ 300 ms) locked to the vocal response and is therefore the consequence of the shifts of the other maps. The topography of map D is congruent with what has been usually found in speech production studies in time-windows preceding the vocal onset beyond 100 ms (see for instance: Laganaro et al. 2012; Valente et al. 2014; Fargier and Laganaro 2016). The topographies of maps B and C (see Fig. 3) have a quite similar configuration characterized by an anterior and very posterior negativity and a central posterior positivity. This microstate has often been reported in the very last time-window of response-locked studies aligned 100 ms before vocal onset but combining ERPs for different onset consonants (see for instance: Bürki and Laganaro 2014; Laganaro 2014; Fargier and Laganaro 2020). Here, these two maps were present in both conditions without statistical difference.

The microstate immediately preceding the vocal onset (map A in Fig. 3) is characterized by a positive activity at frontal and peripheral sites whereas the central electrodes are marked by a more negative activity. The topography of map A is very close to the one reported by Goncharova et al. (2003) in response to frontalis muscle related artifacts, one of the most common source of EMG which is produced by raising eyebrows. Although the eyebrows’ movements were probably limited during the present experiment (participants were told to remain as still as possible even while speaking), a map similar to the one related to frontalis’s muscle may be due to the fact that overt articulation may involve movements from the EEG cap very similar to the ones induced by raising eyebrows. However, the source localization analysis is also consistent with explanation of a specific motor programming/execution origin of the microstate. Indeed, the cerebellum and the premotor cortex/supplementary area were active sources for map A. These different regions are known to be involved in motor sequence learning/execution and, more particularly, they are supposed to be part of the functional network related to motor aspects of speech production (Ackermann et al. 2004; Bohland and Guenther 2006; Riecker et al. 2008). Whatever might be its origin (related to eyebrows raising or speech production), the location of the sources tend thus to confirm the link with the motor system of the microstate A, also given that different activations were found for map D (left temporal activity and a less extended activity in the cerebellum, Fig. 4). We will no further discuss the localization, as neither an individual MRI template, nor 3D digitizing technique for electrode location were used for source localization which may therefore not be precise to warrant further interpretation.

Other ERP studies on oro-facial movement have described similar topography, characterized by a positive anterior circle and a widespread posterior negativity, related to facial or tongue myogenic activity (McMenamin et al. 2011; Ma et al. 2012; Georgieva et al. 2018; Vanhatalo et al. 2003). A similar topography has also been described in speech production studies in late stimulus-locked time-windows, likely associated with articulation (Porcaro et al. 2015; Ouyang et al. 2016). The topography of map “A” hence very likely reflects articulatory movements.

The detailed results of the inter-condition differences further suggest that map “A” reflects the AAI gap. First, its duration is statistically larger for stops than for fricatives, which is in line with the known AAI differences for these phonemes. Second, this 90 ms difference on map duration matches the difference in vocal RTs between plosives and fricatives. Yet, the behavioral significant difference observed between vocal RTs for voiceless fricatives and stops is “virtual” in that it is related to a shorter AAI for /s/ than for /p,t,k/.

The comparison of the results obtained on ERPs averaged per condition in each individual (see “TANOVA and Topographic Pattern Analysis” section) and on single trials (see “Fitting in the Single Trials” section) raises several interesting points. As a matter of fact, similar effects of onset conditions were observed on response latencies and on duration of map A, with a clear effect of mode of articulation (stops vs fricatives) whereas effects of place of articulation were present only on the stop onset consonants. As the AAI is a marker of asynchrony between vocal and articulatory onsets, we were not expecting any particular differences between /sp/, /st/ and /sk/ as they all begin with the same onset consonant and the AAI is quite short for fricatives.

On the other hand, these significant differences in duration of RTs and of map A observed across place of articulation for voiceless stop onset consonants (i.e. between /p/, /t/ and /k/) are in the same directions as previously reported (/k/ < /t/ < /p/ see EAI duration—Table 1 in Rastle et al. 2005) and further suggest that AAI can be identified with ERP microstate analyses.

**Fig. 5:**
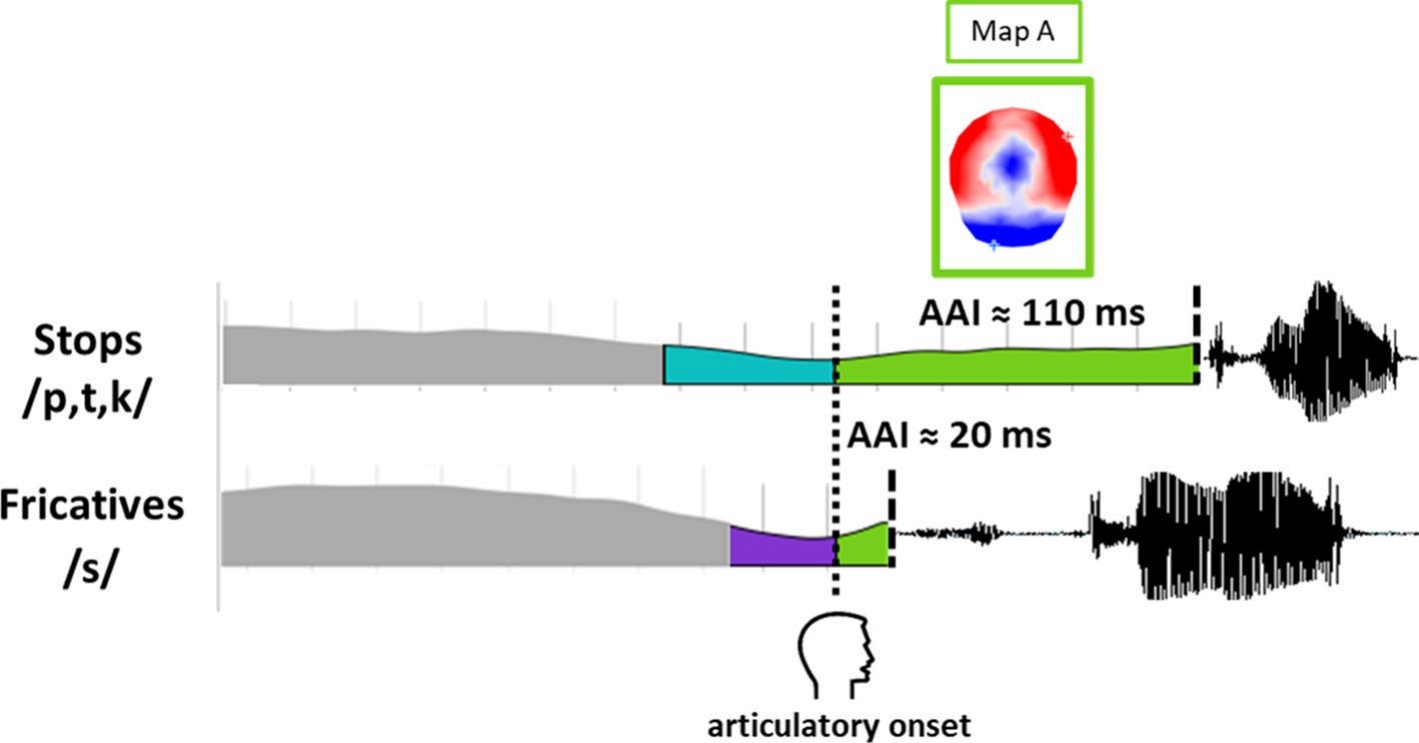
Schematized representation of the shifted AAI depending on initial phoneme

However, compared to the results on single trials, the results based on averaged ERPs (per condition and individual) display a better match with the RT results and also to previously reported AAI values for our onset phonemes in phonetic studies (see similar AAI duration differences between stops and fricatives where behavioural results were also based on averages, for instance Fig. 5. in Mooshamer et al. 2012).

Taking all together, the present results allow us to reasonably claim that the specific microstate corresponding to the topographic map “A” (in Fig. 3 and Fig. 5) is associated with articulation. Its duration reflects the AAI and its onset is therefore well suited to pinpoint onset of articulation. Notice that, as stated in the Introduction, the “onset of articulation” may correspond to three different events (to the onset of movement or to the onset of muscle activity in the absence of movement, or even to the onset of motor execution in the sense of transmission of a motor speech plan to the motor system), but that the present study does not allow us to disentangle these events in case they do not coincide.

Observing different durations across conditions of the period of stable electrophysiological stability corresponding to map A may thus prevent possible misinterpretations of the results on the factors of interest that are manipulated (if they are other than the initial phonemes).

### Conclusion

Overall, the present results confirm the experimental observation that articulation starts several hundred milliseconds before vocal onsets (Halle et al. 1957; Bell-Berti and Harris 1981; Brooker and Donald 1980), and that the duration of the articulatory to acoustic onset (AAI) varies according to the initial phoneme (Rastle et al. 2005; Mooshammer et al. 2012).

Crucially, the results show that a specific ERP microstate covers the known articulatory to acoustic gap for specific onset phonemes and therefore its onset potentially indexes the onset of articulation.

This approach also raises some issues. First it still relies on the vocal onset to detect the articulatory-to-vocal ERP activity and, secondly, ERP results are used to identify a pre-acoustic onset in ERP results (possibly a circularity problem as pointed out by Kriegeskorte et al. 2009). Finally, further investigation would be needed to verify if the results generalize on other initial phonemes.

The present ERP results and their convergence with known articulatory-to-acoustic delays for specific phoneme onsets do however provide guidelines for a visualization of possible AAI differences across conditions and for a better (re-)alignment point of response-locked ERPs. In particular, re-aligning ERPs to the onset of the final microstate -corresponding to the map template “A” in our study-, if it happens to vary across conditions, would avoid misinterpretation of EEG and ERP activity preceding the vocal onset. Such an approach seems promising to study the final stages of speech production as it can help to provide a more precise delimitation between cognitive and motor processes, both necessary to convey a spoken message.

## Electronic supplementary material

Below is the link to the electronic supplementary material.

Supplementary file1 (DOCX 137 kb)

## Footnotes

1 Here we will refer to the notion of AAI introduced by Kawamoto et al. (2008), although other authors used different terminologies (e.g. Rastle et al. 2005).

2 The stimuli used here are part of a larger protocol, aimed also to test the effect of syllabic complexity (structure and legality) on speech planning. The results presented in this work are limited to the analysis of the phoneme onset, this is why all manipulations of stimuli will not be considered here.

3 As the main interest of this paper is to study the AAI which happens during the last phases of speech production, only the response locked analysis are presented here. See Supplementary material for stimulus-locked ERPs analysis.

4 One TF being approximatively equivalent to 2 ms (1.96 ms).
