# Supplementary material for: Microstate ERP Analyses to Pinpoint the Articulatory Onset in Speech Production
Source: Brain Topogr. 2020 Nov 8;34(1):29–40. doi: 10.1007/s10548-020-00803-3 (PMC7803690; doi:10.1007/s10548-020-00803-3)
Supplement: Supplementary file 1 — Supplementary file1 (DOCX 137 kb) [file 10548_2020_803_MOESM1_ESM.docx]

**Supplementary material / Appendix**

- **Appendix 1:** **Stimulus-locked analysis**

The pre-processing and analysis were similar to the ones described for response-locked data, but were locked to the question mark eliciting the delayed overt production.

Pairwise TANOVAs on the stimulus-locked ERPs revealed no significant differences across conditions.

The spatio-temporal segmentation applied on the grand-averaged data for each condition revealed five different electrophysiological template maps for the stimulus-locked ERPs. The maps 1 to 5 for the stimulus-locked ERPs were fitted from 0 to 150 TF.


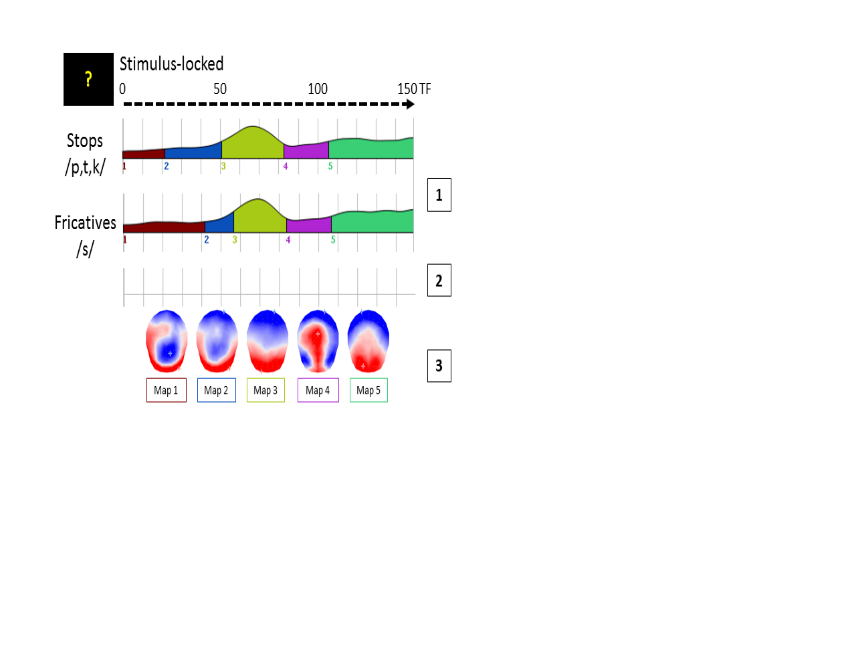


Figure S1. (1)Temporal distribution of the topographic maps revealed by the spatio-temporal segmentation: analysis displayed on the mean GFP from the grand average of each condition locked to stimulus, with the corresponding template maps 1,2,3,4 and 5 (3). (2) TANOVA show no significant results.

The micro-states segmentation results globally showed little differences between the two initial-phoneme conditions for the stimulus-locked ERPs. Statistical differences (in duration and GEV, see Table S1) were observed on the two first maps (1 and 2) which precede the P100 (map 3). The maps 1 and 2 are supposed to be related to the “base time”, i.e. time to detect the response signal. No other differences were found after the P100 elicited by the question mark and the TANOVA did not yield topographic differences, meaning that the earliest processes occurring after the cue for the delayed production are common for voiceless stops and fricatives.

Table S1: Duration (in number of TF) and Global Explained Variance (in %) for the maps of the topographic pattern analysis (stimulus-locked analysis)

|  | | **Duration (Number of TF)** | | | | | **GEV (%)** | | | | |
| --- | --- | --- | --- | --- | --- | --- | --- | --- | --- | --- | --- |
| Comparison | | Mean | Std. error | df | t | p value | Mean | Std. error | df | t | p value |
| Map 1 | Stop  Fricatives | 22.67  34.05 | 5.52  5.82 | 21 | 2.09 | .02 | 0.78  1.51 | .002  .003 | 21 | 2.09 | .02 |
| Map 2 | Stop  Fricatives | 27.76  14.71 | 3.86  3.26 | 21 | 2.09 | .002 | 2.96  1.49 | .006  .003 | 21 | 2.09 | .01 |
| Map 3 | Stop  Fricatives | 39.00  41.62 | 4.35 5.26 | 21 | 2.09 | .39 | 16.49  16.83 | .02  .02 | 21 | 2.09 | .85 |
| Map 4 | Stop  Fricatives | 26.57  29.29 | 4.26  4.86 | 21 | 2.09 | .16 | 1.80  1.90 | .004  .004 | 21 | 2.09 | .62 |
| Map 5 | Stop  Fricatives | 34.00  31.71 | 5.69  5.56 | 21 | 2.09 | .32 | 9.10  9.37 | .02  .01 | 21 | 2.09 | .81 |
